# Metagenomic analysis of Mesolithic chewed pitch reveals poor oral health among stone age individuals

**DOI:** 10.1038/s41598-023-48762-6

**Published:** 2024-01-18

**Authors:** Emrah Kırdök, Natalija Kashuba, Hege Damlien, Mikael A. Manninen, Bengt Nordqvist, Anna Kjellström, Mattias Jakobsson, A. Michael Lindberg, Jan Storå, Per Persson, Björn Andersson, Andrés Aravena, Anders Götherström

**Affiliations:** 1 Department of Biotechnology, Faculty of Science, Mersin University, 33100 Yenişehir Mersin, Turkey; 2 Department of Archaeology and Ancient History, Uppsala University, Engelska Parken, Thunbergsvägen 3H Box 626, 751 26 Uppsala, Sweden; 3 Museum of Cultural History, University of Oslo, St. Olavs Plass, P.O. Box 6762, NO-0130 Oslo, Norway; 4 PAES, Ecosystems and Environment Research Programme, Faculty of Biological and Environmental Sciences and Helsinki Institute of Sustainability Science, University of Helsinki, Viikinkaari 1, P.O. Box 65, Helsinki, Finland; 5 Foundation War-Booty Site Finnestorp, Klarinettvägen 75, 434 75 Kungsbacka, Sweden; 6 Department of Archaeology and Classical Studies, Osteoarchaeological Research Laboratory, Stockholm University, Stockholm, Sweden; 7 Department of Organismal Biology, Human Evolution, Uppsala University, Evolutionsbiologiskt Centrum EBC Norbyvägen 18 A, Uppsala, Sweden; 8 Department of Chemistry and Biomedical Sciences, Faculty of Health and Life Sciences, Linnaeus University, Hus Vita, 44018 Kalmar, Sweden; 9 Department of Cell and Molecular Biology (CMB), Karolinska Insitutet, P.O. Box 285, 171 77 Stockholm, Sweden; 10 Department of Molecular Biology and Genetics, Faculty of Science, Istanbul University, Vezneciler, 34134 Istanbul, Turkey; 11 Centre for Palaeogenetics, Svante Arrhenius Väg 20C, 106 91 Stockholm, Sweden; 12 Department of Archaeology and Classical Studies, Archaeological Research Laboratory, Stockholm University, Stockholm, Sweden

**Keywords:** Palaeoecology, Archaeology, Microbial communities, Pathogens

## Abstract

Prehistoric chewed pitch has proven to be a useful source of ancient DNA, both from humans and their microbiomes. Here we present the metagenomic analysis of three pieces of chewed pitch from Huseby Klev, Sweden, that were dated to 9,890–9,540 before present. The metagenomic profile exposes a Mesolithic oral microbiome that includes opportunistic oral pathogens. We compared the data with healthy and dysbiotic microbiome datasets and we identified increased abundance of periodontitis-associated microbes. In addition, trained machine learning models predicted dysbiosis with 70–80% probability. Moreover, we identified DNA sequences from eukaryotic species such as red fox, hazelnut, red deer and apple. Our results indicate a case of poor oral health during the Scandinavian Mesolithic, and show that pitch pieces have the potential to provide information on material use, diet and oral health.

## Introduction

The Scandinavian Peninsula (hereafter referred to as Scandinavia) gradually became accessible to humans when the Weichsel ice sheet melted away after the late glacial maximum (LGM) peaked between 27,000 and 21,000 years before present (BP). There is material evidence for sporadic human presence in ice-free areas around 16,000 BP, but from around 12,800 BP there was a proper expansion into Scandinavia^1^, and from 11,700 BP there was a continuous human presence in the southwestern part of the Peninsula^2–4^. However, the earliest human DNA from the Scandinavian Peninsula is only slightly younger than 10,000 years old^5^.

The early Mesolithic sites exhibit a rich material culture (lithics, bone and antler) that has been used to infer demography, mobility, social relations, use of technology, and the subsistence strategies^6^. While this has provided an understanding of the Early Mesolithic people in Scandinavia, we still don’t have enough molecular data to contextualise their oral microbiome profile, pathogen burden, diet related information, and paleoenvironmental utilisation.

Recently, metagenomic analysis of ancient DNA reads sequenced from ancient human samples have enabled researchers to obtain information about the historical human populations with a great depth. Ancient dental calculus has become the predominately used material to investigate oral microbiome, due to its structural integrity^7^. Lately, chewed pitch materials are also proved to be useful to access historical oral microbiome^8^.

Ancient dental calculus studies provide information about the oral microbial species in dental biofilm formation and maturation, and its association with dental health^9–11^. Moreover, changes in the oral microbiome through archaeological time periods can also be investigated. For example, the transition from hunter-gatherer societies to farmers brought a significant increase in human oral health pathologies, which are associated with dietary and lifestyle changes^12^. Several ancient DNA studies had investigated changes in oral microbiome composition from the Mesolithic period to modern day and their relation to dietary changes and population movements^13–16^, as well as pre and post Columbian contact^17^. It is also possible to identify dietary remains and DNA reads related to the paleoenvironment through ancient DNA and microscopic studies^18–20^.

Another promising source of ancient microbiome DNA investigated here are masticates, or pitch pieces that have been chewed by humans and therefore contain materials from the oral cavity. Previous studies had investigated the oral microbiome and dietary components from 5,700 years old chewed pitch from Denmark and successfully proved the potential usage in ancient DNA research domain^5,8^.

To better understand the Mesolithic community in Huseby Klev, we provide a metagenomic analysis of the pitch mastic pieces that are more than 9,500 years old, and that already have yielded information on Mesolithic demography^5^. This DNA represents the oldest known genomic and metagenomic material to date from humans and human activities in Scandinavia.

Our analysis shows the oral microbiome profile in three chewed pitch pieces along with high coverage genomes of human commensal oral microbes. By comparing our data with modern oral microbiomes, we demonstrate the increased abundance of oral microbes that could cause pathological conditions such as periodontitis. Moreover, we provide DNA evidence on dietary components and possible paleoenvironmental eukaryotes that represents the Huseby Klev archaeological site^21^.

## Results

### A snapshot of the Mesolithic oral microbiome

To have a broad overview of the metagenomic composition in our ancient pitch materials, we first compared the metagenomic profile of the samples (ble004, ble007, and ble008) against the taxonomic composition reported in the modern human oral cavity (*N*  = 725), modern human nasal cavity (*N*  = 93), modern human skin (*N*  = 42), modern human stool (*N*  = 500), modern human vagina (*N*  = 86), ancient human dental calculus (*N* = 395), and chewed pitch (N = 1) samples. The last sample is also chewed pitch but from a later period (5,858–5,661 cal. BP)^8–11,14–17,19,22–31^.

Phylum profiles of the ancient samples show a pattern closely related to oral microbiome samples. Also, the phylum-level profile of the pitch samples closely resembles the Syltholm pitch profile (Figure S1). In addition, the species' relative abundances show a remarkable presence of commensal human bacteria (Figure S2).

**Figure 1:**
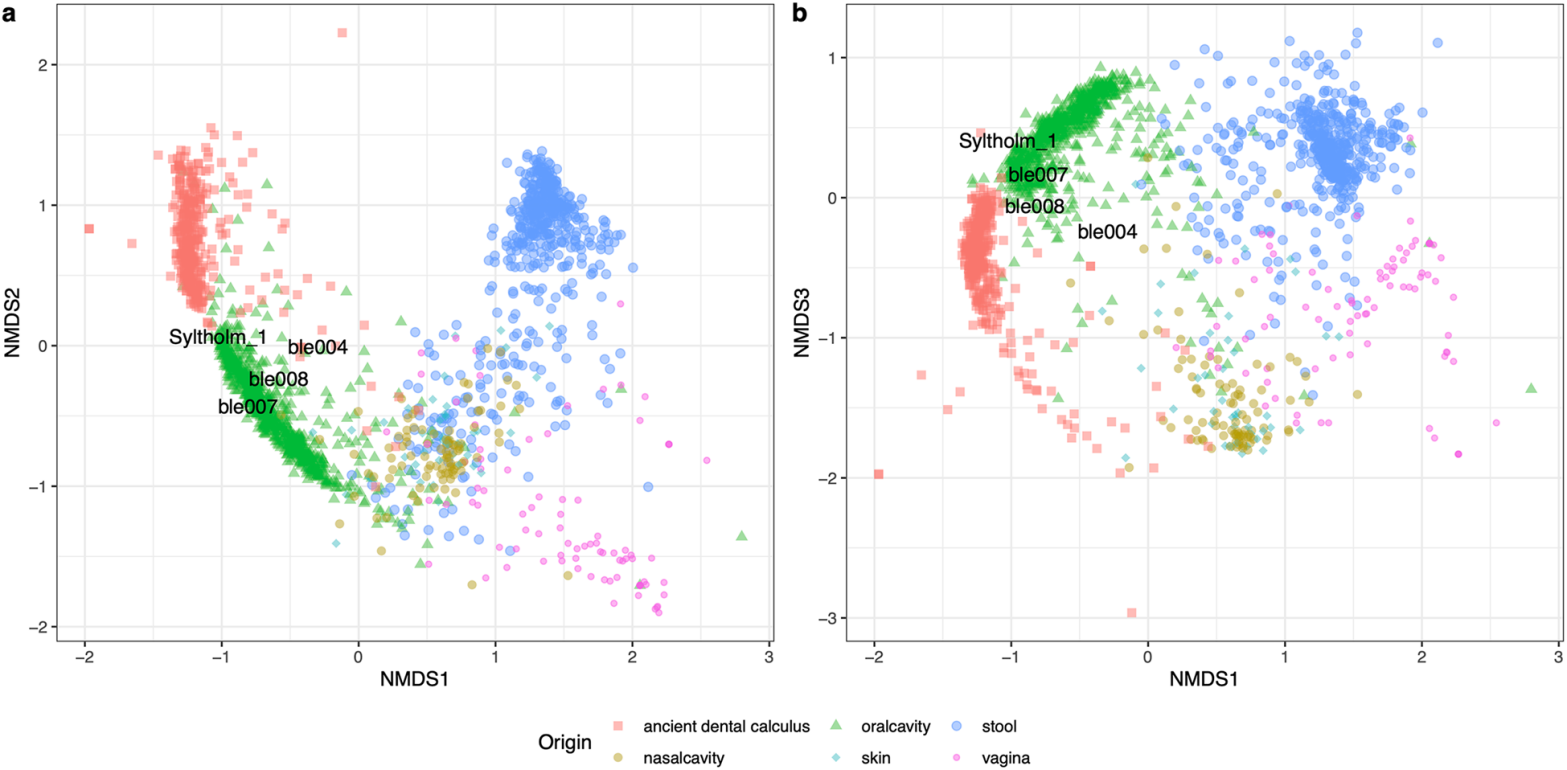
Non-metric multidimensional projection of the Bray–Curtis distance matrix based using the MetaPhlAn3 species abundance matrix. In this figure, we used in modern human oral cavity (*N*  = 725), modern human nasal cavity (*N* = 93), modern human skin (*N* = 42), modern human stool (*N*  = 500), modern human vagina (*N*  = 86), ancient human dental calculus (*N*  = 395), and chewed pitch (*N*  = 1) to characterise the microbiome composition in the ancient chewed materials. We can see that ancient chewed pitch materials are similar to the oral samples.

Subsequently, we calculated Bray–Curtis distances using species taxonomic composition to compare microbial diversity between the samples^32^ and visualised the distance matrix with the non-Metric Multidimensional Scaling (NMDS) method. The NMDS plot and Bray–Curtis distances show that microbial compositions in the ancient pitch material are closer to oral samples than to other parts of the human body. The ancient ble pitch samples cluster with modern oral microbiome samples in the NMDS plot along with the Syltholm chewed birch pitch (Fig. 1, Figure S3). In addition to these analyses, we quantified the potential oral microbiome contribution to the ancient mastic samples by using the same external dataset along with environmental sediment samples and we observed 39—57% of oral microbiome and ancient dental calculus contribution to ble chewed pitch pieces (Figure S4) ^33–36^.

These results strongly suggest that the microbial profile of the pitch material originates from the oral cavity, corroborating that the mastic pieces were orally processed.

### Validation of bacterial species presence

The taxonomic profiling mapped DNA reads in our libraries to 281 bacterial species (Table S1). To validate the presence of these bacteria in our ancient chewed pitch material, we aligned all reads to their corresponding reference genome. A homogeneous coverage across the genomes distinguishes bacterial presence from false positives.

We found 51 individual bacterial genomes with a minimum of 1 × depth of coverage and 40% breadth of coverage (Table 1). Most of these genomes correspond to pathogenic or opportunistic oral bacterial species. For example, we identified sequences from *Porphyromonas gingivalis* and *Treponema denticola* that belong to the red complex (a group of species that are found predominantly in periodontitis cases), *Aggregatibacter actinomycetemcomitans* (predominantly in sample ble004), and *Streptococcus mutans*. Furthermore, in all samples and predominantly in sample ble004 we note the presence of *Haemophilus influenzae*, *Aggregatibacter actinomycetemcomitans, Cardiobacterium valvarum*, *Eikenella corrodens*, and *Kingella denitrificans*.

**Table 1:** Genome reconstruction of ancient pathogens. Single stranded deamination rates were low for ble004 samples since damage-repaired libraries were included in the analysis. However, higher deamination rates observed in non-damage repaired ble004 DNA libraries confirm their authenticity. DOC: Mean depth of coverage, BOC: Breadth of coverage, δS: Single stranded deamination rate, SD: Standard deviation for delta S.

Sample	Bacteria	Read Count	Mean length	DOC	BOC (%)	δS	SD
ble004	*Rothia dentocariosa* ATCC 17,931 X	802 082	52.90	16.93	92	0.20	0.01
ble004	*Corynebacterium durum* F0235	253 338	50.83	4.58	91	0.33	0.03
ble004	*Lautropia mirabilis* ATCC 51,599	1 196 572	50.07	18.96	90	0.99	0.01
ble004	*Actinomyces johnsonii* F0542	535 182	50.93	8.19	90	0.93	0.06
ble004	*Rothia mucilaginosa* DY-18	1 446 426	53.05	33.84	87	0.98	0.02
ble004	*Abiotrophia defectiva* ATCC 49,176	792 762	52.25	20.27	86	0.14	0.00
ble004	*Gemella sanguinis* ATCC 700,632	543 902	60.69	18.38	85	0.18	0.01
ble004	*Actinomyces oris*	600 330	48.90	9.65	85	0.80	0.15
ble004	*Pseudomonas fluorescens* F113	680 499	84.30	8.38	85	0.12	0.23
ble004	*Streptococcus sanguinis* SK36	623 791	53.65	14.01	83	0.12	0.00
ble004	*Eubacterium nodatum* ATCC 33,099	178 607	55.68	5.43	81	0.15	0.01
ble004	*Granulicatella adiacens* ATCC 49,175	376 560	54.45	10.53	81	0.12	0.00
ble004	*Peptostreptococcus stomatis* DSM 17,678	301 582	58.23	8.83	80	0.13	0.00
ble004	*Neisseria mucosa* C102	1 227 593	54.03	30.75	79	0.15	0.00
ble004	*Streptococcus gordonii* str. Challis substr. CH1	245 194	54.45	6.08	79	0.13	0.01
ble004	*Neisseria elongata* subsp. glycolytica ATCC 29,315	254 896	52.56	5.94	77	0.22	0.01
ble004	*Veillonella parvula* DSM 2008	168 862	53.53	4.24	63	0.13	0.01
ble004	*Parvimonas micra*	120 248	56.76	4.19	59	0.14	0.01
ble004	*Streptococcus thermophilus* JIM 8232	196 068	52.48	5.33	51	0.09	0.01
ble004	*Streptococcus salivarius*	247 717	52.05	5.89	46	0.09	0.00
ble007	*Rothia dentocariosa* ATCC 17,931	71 783	64.89	1.86	71	0.64	0.01
ble007	*Rothia mucilaginosa* DY-18	115 316	61.53	3.13	67	0.65	0.01
ble007	*Gemella sanguinis* ATCC 700,632	62 974	70.56	2.47	67	0.73	0.02
ble008	*Rothia dentocariosa* ATCC 17,931	107 407	71.61	3.07	80	0.51	0.01
ble008	*Corynebacterium durum* F0235	76 522	70.79	1.93	76	0.53	0.02
ble008	*Eggerthia catenaformis* OT 569 = DSM 20,559	41 568	NA	1.82	72	0.57	0.02
ble008	*Rothia mucilaginosa* DY-18	125 238	66.01	3.65	71	0.55	0.01
ble008	*Lautropia mirabilis* ATCC 51,599	95 446	68.10	2.06	65	0.66	0.02
ble008	*Gemella sanguinis* ATCC 700,632	45 607	80.51	2.04	64	0.56	0.03
ble008	*Streptococcus sanguinis* SK36	72 704	69.91	2.13	61	0.40	0.01
ble008	*Streptococcus mutans* UA159	25 788	79.79	1.01	59	0.52	0.02
ble008	*Neisseria mucosa* C102	52 113	70.37	1.69	57	0.49	0.02
ble008	*Abiotrophia defectiva* ATCC 49,176	41 683	67.71	1.38	52	0.52	0.02
ble008	*Gemella haemolysans* ATCC 10,379	26 027	77.24	1.05	49	0.39	0.02
ble008	*Streptococcus salivarius*	51 019	67.50	1.57	46	0.45	0.02
ble008	*Streptococcus mitis* B6	40 508	67.63	1.28	43	0.42	0.02
ble008	*Granulicatella adiacens* ATCC 49,175	31 470	69.88	1.13	42	0.45	0.03
ble008	*Streptococcus thermophilus* JIM 8232	47 626	67.62	1.67	41	0.41	0.02

### Authentication of ancient oral bacteria

We used three established criteria to authenticate the antiquity of the reads^37,38^: edit distance distribution, length distribution, and deamination patterns. It is expected that most of the sequences have edit distance at most 1. Also, we expect the majority of the sequences to be shorter than 100 nucleotides, and they should have deamination patterns towards the ends (Table S1, Figure S5). Edit distance describes the number of substitutions between a reference genome and aDNA reads thus providing an estimate for the evolutionary distance.

Table 1 shows the summary statistics after the genome alignment steps with at least 1 × depth of coverage. Note that several libraries of the ble004 sample were damage-repaired for human population genetics analysis, and thus shows a lower deamination rate. However, the non-damage repaired libraries of ble004 show the authentic deamination patterns.

### De novo assembly of ancient bacteria

The metagenomic profile indicates presence of ancient oral bacteria, several of them likely opportunistic pathogens. Next, we reconstructed large portions of the ancient genomes by assembling de novo to further authenticate our data (Table S2). This strategy enabled us to assemble contigs that could be associated to a total of 49 unique bacterial species in our data.

### Differentially abundant oral bacteria in ancient pitch materials

The types of bacterial species present in ancient chewing gum appears to be useful to explore the oral microbiome. This is in concordance with recent studies where some 15% of the salivary microbiome seems to get encapsulated in modern chewing gums^39^. Thus, the microbiome profiles from the pitch material should make it possible to identify the signs of dysbiosis in the microbiomes of the ancient individuals.

**Figure 2:**
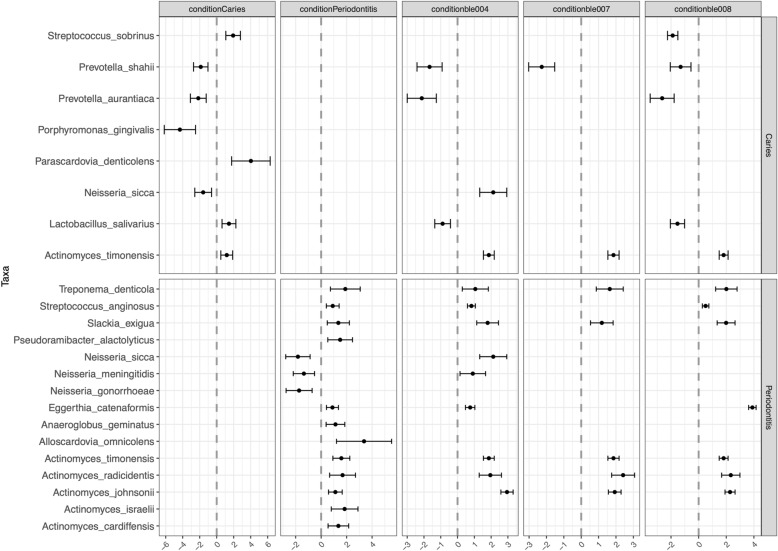
The Beta Binomial method applied to ancient mastics. Rows (Caries and Periodontitis) describing the two dysbiosis conditions tested. Each column represents a comparison condition. The condition control column represents the marker species that are significantly changed in each dysbiosis condition. Condition ble004, ble007 and ble008 columns represent the microbial species that were significant changes in ancient materials, compared to healthy cases.

Using modern periodontitis, caries, and healthy salivary datasets^40,41^, we explored the presence of differentially abundant microbes that are specific to dysbiosis conditions. The analysis indicated 27 differentially abundant bacteria that could be used as markers for periodontitis and caries conditions (Fig. 2, Table S3). We found increased abundances of *Actinomyces* species along with *Treponema denticola*, *Streptococcus anginosus*, *Slackia exigua,* and *Fusobacterium nucleatum,* which are common in modern periodontitis cases. Concerning dental caries, we found increased abundance of *Streptococcus sobrinus* and *Parascardovia denticolens* compared to healthy oral microbiome profile.

With this method, we identified differentially abundant bacterial species in libraries ble004, ble007, and ble008 (Table S4). Several of those marker species were found in ancient masticated pitch samples such as *Treponema denticola*, *Actinomyces johnsonii*, *Actinomyces timonensis*, and *Streptococcus anginosus* (Table 2).

**Table 2:** Differential abundance of the marker species in BLE samples using the beta-binomial method. The taxa with increased differential abundance in the marker set is shown in bold. Markers: the taxa that we found differentially abundant in dysbiosis conditions.

	Species	Markers	ble004	ble007	ble008
Periodontitis	***Treponema_denticola***	**1.9 ± 1.2**	**1.06 ± 0.8**	**1.64 ± 0.8**	**2.01 ± 0.8**
	***Actinomyces_israelii***	**1.84 ± 1.1**	**–**	**–**	**–**
	***Actinomyces_timonensis***	**1.59 ± 0.7**	**1.86 ± 0.3**	**1.86 ± 0.3**	**1.81 ± 0.3**
	***Actinomyces_johnsonii***	**1.11 ± 0.5**	**–**	**1.93 ± 0.4**	**2.27 ± 0.4**
	***Streptococcus_anginosus***	**0.9 ± 0.5**	**0.83 ± 0.2**	**–**	**0.5 ± 0.2**
	***Eggerthia_catenaformis***	**0.89 ± 0.5**	**0.75 ± 0.3**	**–**	**3.89 ± 0.3**
	*Neisseria_sicca*	−1.82 ± 0.9	2.13 ± 0.8	**–**	**–**
Caries	***Streptococcus_sobrinus***	**1.94 ± 0.9**	**–**	**–**	**−1.88 ± 0.4**
	*Prevotella_shahii*	−1.88 ± 0.9	−1.67 ± 0.7	−2.27 ± 0.7	–
	*Prevotella_aurantiaca*	−2.17 ± 0.9	−2.13 ± 0.9	–	−2.64 ± 0.9
	*Porphyromonas_gingivalis*	−4,33 ± 1.8	–	–	–

Moreover, the results suggest a significant increase in the oral commensal bacterial species, in respect to healthy salivary samples such as *Rothia dentocariosa*, *Gemella sanguinis*, *and Streptococcus sanguinis* as well as *Haemophilus influenzae*, *Aggregatibacter actinomycetemcomitans, Cardiobacterium valvarum*, *Eikenella corrodens*, and *Kingella denitrificans*.

The ble004 sample is especially interesting, as eleven periodontitis-specific marker species show abundance patterns linked to disease. On the other hand, we found only one marker species (*Actinomyces timonensis*) linked to dental caries.

### Machine learning predicts periodontitis-like oral microbiome in chewed pitch materials

We used a machine learning method to find the species that correlate with dysbiosis, and to predict sign of dysbiosis in ancient samples.

Using modern salivary microbiome data, we trained several Random Forests^42^ models for periodontitis and caries conditions, and selected the features (species) that contribute to the overall accuracy of the models. The best models classified the correct health status of the samples with an estimated error rate under 14%. The selected models identified 38 bacterial species (Table S6 for the periodontitis and caries models. They included most of the marker species that are differentially abundant in dysbiosis cases, and several additional species.

**Figure 3:**
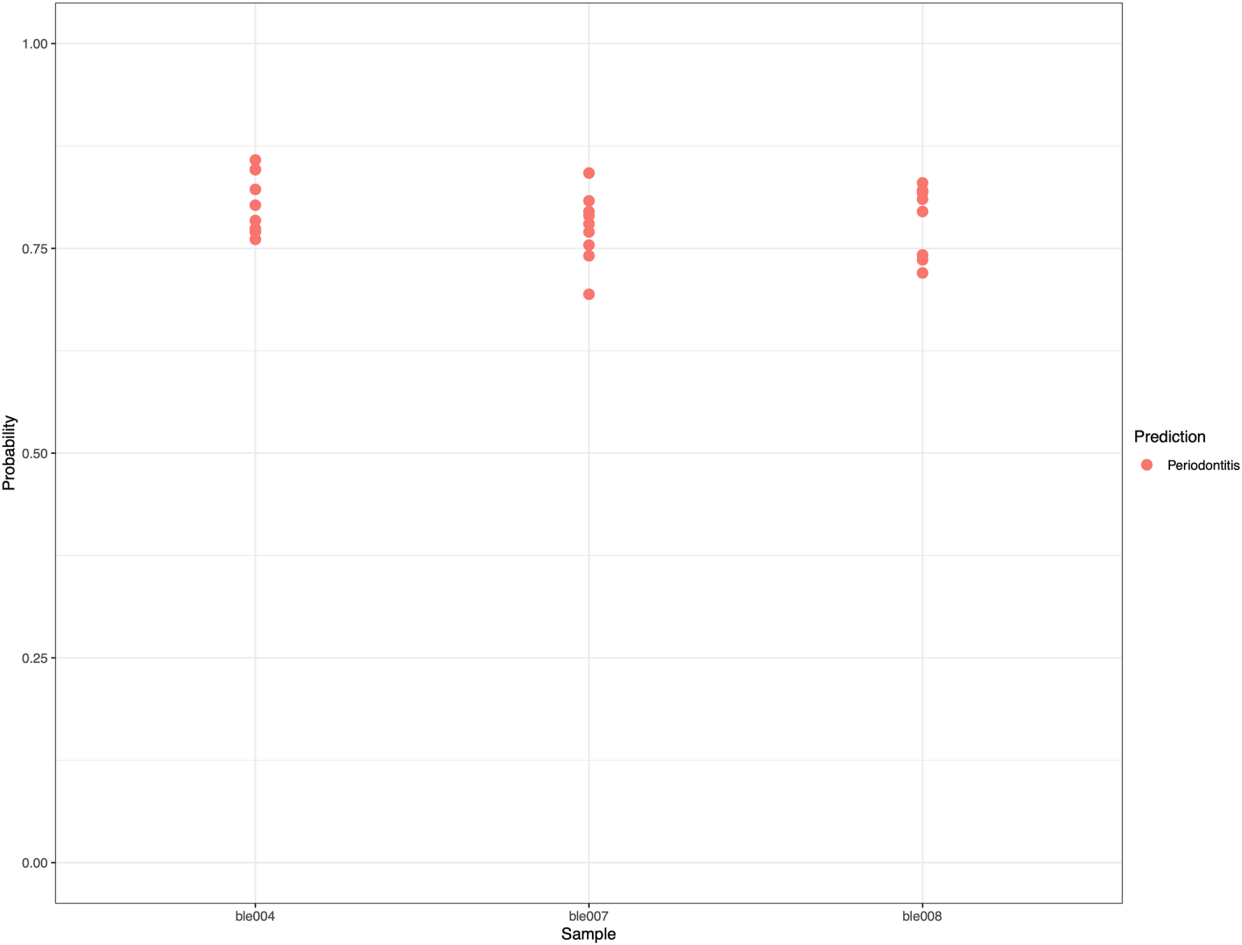
The class prediction probability values from at least six different best models were combined and the predictions are shown for Periodontitis models.

The clear results, including the low estimated error rates, allow us to infer the microbiome profile in ancient pitch materials. This was carried out by comparing the microbial abundances in the periodontitis and dental caries models, and calculating the probability values for concordance (Fig. 3). The probability values for the dental caries models were not high enough to conclude the presence of caries with sufficient certainty. However, the periodontitis models suggest the occurrence of periodontitis based on the oral microbiome from ancient pitch samples, with more than 70% probability. In the ble004 sample, the probability of the presence of a periodontitis-like microbiome is 84%. These results supported the conclusions from the differential abundance analysis.

### Eukaryotic species in ancient pitch pieces

Using five Kraken2 confidence interval thresholds, we authenticated DNA reads from 15 possible eukaryotic species (Table S7—S11). In Table 3, only results from threshold 0.5 are shown. DNA reads mapped to hazelnut (*Corylus avellana*), red deer (*Cervus elaphus*), and brown trout (*Salmo trutta*) in ble004 and ble007; wolf (*Canis lupus)*, hazelnut, red fox (*Vulpes vulpes*), arctic fox (*Vulpes lagopus*) in ble008 were present in all five threshold values. Eelgrass (*Zostera marina*) in ble004 and mallard (*Anas platyrhynchos*) in ble007 were present in four thresholds. Wolf, European robin (*Erithacus rubecula*), European crab apple (*Malus sylvestris*) in ble004 and European turtle dove (*Streptopelia turtur*) were present in three thresholds. Moreover, we authenticated DNA reads from mistletoe (*Viscum album*), apple (*Malus domestica*), tufted duck (*Aythya fuligula*), and limpet (*Patella vulgata*) in other Kraken2 thresholds.

**Table 3:** Authenticated eukaryotes using Kraken2 confidence interval threshold of 0.5.

Sample	Taxid	Latin name	English name	Reads after Processing	Mean Read length
ble004	13,451	*Corylus avellana*	Hazelnut	30,038	54.98
ble004	37,610	*Erithacus rubecula*	European robin	4209	50.48
ble004	9860	*Cervus elaphus*	Red deer	1942	56.41
ble004	296,552	*Zoster marina*	Eelgrass	913	68.44
ble004	8032	*Salmo trutta*	Brown trout	825	71.28
ble007	8839	*Anas platyrhynchos*	Mallard	172,172	65.23
ble007	1,771,552	*Streptopelia turtur*	European turtle dove	3348	66.34
ble007	8032	*Salmo trutta*	Brown trout	3164	69.40
ble008	9612	*Canis lupus*	Wolf	1,294,009	76.48
ble008	13,451	*Corylus avellana*	Hazelnut	177,612	79.29
ble008	9627	*Vulpes vulpes*	Red fox	5633	69.33
ble008	494,514	*Vulpes lagopus*	Arctic fox	4318	67.65

These species passed the visual confirmation criteria: edit distance, length distribution, and deamination patterns.

In particular, wolf and hazelnut in ble008, mallard in ble007, and hazelnut in ble004 samples had more than 10,000 reads aligned to the respective reference sequences. Moreover, we found 1059 reads mapped to the mitochondrial genome of red fox in the ble008 sample.

## Discussion

To broaden our understanding of human health and everyday life in Mesolithic Scandinavia, we investigated the metagenomic content of three pieces of ancient pitch material from one site. Using this data, we explored oral microbiome composition, diet related DNA reads, and resource utilisation.

The non-metric multidimensional scaling on Bray-Curtis^32^ distances grouped chewed pitch materials close to human oral microbiome and ancient dental calculus samples. Also, *SourceTracker*^43^ analysis showed a contribution of around 50% of modern oral microbiome and ancient dental calculus to ancient pitch samples. Moreover, there is a high relative abundance of species that are known to be commensal oral bacteria. This suggests that the DNA from chewed ancient pitch pieces is not specific to the pitch, nor to a specific tissue, but to most of the oral area in which the pitch was chewed.

To validate the presence of these commensal oral bacterial species we used two approaches. First, we compiled a large database curated from full length genomic sequences of Archaea, Bacteria, Viruses and Fungi, and used *malt*^38^ (an alignment-based metagenomic classification method) to confirm the presence of known oral commensal bacteria. Also, we de novo assembled the DNA reads and created draft ancient bacterial genomes. Therefore, our analysis proves that the metagenomic content in ancient pitch pieces is closer to oral microbiome profile and contains commensal oral bacterial species.

A general problem with ancient microbial paleopathology is that there can be bacterial presence without physical symptoms. Generally, the presence of pathogenic species correlates with dysbiotic states but not necessarily in a causal way. Thus, our results suggest dysbiosis but we cannot show disease symptoms.

Subsequently we examined the microbial species in pitch pieces that are over abundant. To do this, we used a modern salivary microbiome dataset that contains healthy, periodontitis, and dental caries conditions and ancient dental calculus samples that were identified as periodontitis.

Recently, Nearing et al.^44^ tested several differential abundance tools and showed a large variability between methods. Thus, they suggest using a consensus, that is, i. e. on results where several tools (based on different methods) agree.

In our study we applied two methods to identify bacterial species that correlate with dysbiosis conditions: a statistical method based on the beta-binomial distribution, and machine learning using Random Forests. The logic is, as suggested by Nearing et al.^44^, that the agreement between several techniques is a stronger argument for the detection of a given condition. Along the same line of reasoning, the reliability for dysbiosis detection increases when it is detected by several methods.

Using the modern salivary microbiome datasets, we identified specific bacterial species that are correlated with dysbiosis conditions and we search for the presence of these species in ancient chewed pitch pieces.

When we compare healthy and periodontitis modern samples, we observe increased abundance of *Streptococcus*, *Actinomyces*, *Eubacterium*, *Dialister*, and *Treponema* species along with a reduction of abundance of *Neisseria,* (Fig. 2) which is coherent with the findings of Li et al^45^.

*Streptococcus mutans* has been the most studied marker for dental caries, even though it is not considered to be the only cause of dental caries. For example, even in the absence of *Streptococcus mutans*, several acidophilic bacteria (e.g. *Lactobacillus* species) have been shown to co-occur with dental caries^46,47^.

Previous studies have shown that *Treponema denticola*, *Slackia exigua*^48^, *Eubacterium brachy*, and *Streptococcus anginosus* are likely associated with periodontitis. The predictor bacterial species from the best random forest models in this study identified most marker species and added additional potential markers. Moreover, in the caries random forest models, we found the marker *Streptococcus mutans*, which has been extensively studied in connection with dental caries cases. This is in line with the results of the beta-binomial test.

We confirmed the presence of the marker species in the ancient pitch material. We observed an increased abundance of periodontitis characteristic bacteria in all samples. Moreover, random forests models predict high probability for periodontitis in the Mesolithic individuals who chewed the pitch pieces. This is especially clear in ble004, where we had the highest number of differentially abundant markers.

The list of bacteria from the Syltholm chewed pitch material, which is a type of material similar to ours, indicates the presence of periodontitis specific bacterial taxa from the red-complex species. This sample dates to the Neolithic transition period but the Mesolithic human genetic component is compatible with our material^8^.

When contextualised, the results are compatible with what is generally known about the Mesolithic Scandinavian society. The Mesolithic population density was low^49^, with limited possibilities for pandemic-causing microbes to spread between humans, but not restricting the presence of bacteria from other sources than humans, like for example those causing systemic diseases including infective endocarditis. The wider use of the teeth, as tools, likely increased the risk for collecting periodontitis causing oral microbes.

The genetic traces we found complements the osteological and palaeobotanical results found from Huseby Klev and the species list in Table 3 reflects the palaeoenvironment of a settlement site located in the transitional zone between terrestrial and marine ecosystems. In particular, the remains of wolves, red fox, hazelnut, red deer and mallard were found in the several layers of Huseby Klev. In this perspective, the genetic data is consistent with the archaeological remains on site and reflects a Mesolithic environment^21,50^.

Moreover, other eukaryotic remains also fit the description of other Mesolithic sites in Europe. For example, hazelnut shells are frequently found at contemporary sites^1,8,51^. Also, reports show that limpets could be a dietary component in the Mesolithic period to complement meat from ungulates^52^. Bird bones were commonly used to produce tools in the Mesolithic. DNA sequences from Tufted duck, European robin, and mallard could therefore derive from birds used as food and/or raw material.

Even though remains of salmonids were not present, the presence of other species like cod (*Gadus morhua*) indicates that fishing was part of the subsistence strategy^21^. It has been suggested that salmonids are usually not detectable due to poor fish bone preservation^53^. Moreover, reference genomes for fishes are mostly not complete. Therefore, it is possible that the DNA reads assigned to trout because of scanty reference genomes.

Mistletoe is a hemiparasitic plant that infects hard and softwood trees. It is frequently found in Europe and often discovered by macroscopic analysis at Mesolithic sites in central Europe. Reports suggest that the plant could be used to produce poison for arrowheads, in addition to being utilised for medicinal purposes^54^. Crab apple pieces, cores and wood from apple trees are also found at the site^41^ as in several Mesolithic sites, and it is native to Europe.

In sample ble008, red fox, arctic fox and wolf were identified. These all three are members of the *Canidae* family, and they share a common ancestor. Thus separating these three species can be problematic. The presence of red fox is consistent with Mesolithic Scandinavian fauna, and their presence in the pitch samples is perhaps surprising but still consistent with osteological material from Huseby Klev. Furs of *Canidae* were probably used for clothing and the meat may have been consumed. Even though recent reports have demonstrated hunting of arctic foxes in Southern Poland 25,000 years ago^55^, the climate around Huseby Klev was too mild (annual mean temperature was 8℃) for this species to live^56^. We therefore used a competitive mapping approach and confirmed the presence of the mitochondrial genome of the red fox with 31% breadth of coverage.

Boethius et al.^21^ analysed strontium content of faunal and floral remains from Huseby Klev to trace evidence of mobility. The authors suggest that the residents of Huseby Klev were mobile, and used waterways to travel distances up to 20 km for hunting. The presence of terrestrial animal DNA in the pitch material indicates hunting of those species which is in agreement with the osteo-archaeological material from the Huseby Klev site.

Recently Mann et al.^18^ demonstrated the challenges in identifying eukaryotic reads in ancient samples. In essence, the relatively low number of aDNA sequences, and shared evolutionary ancestry in eukaryotic species create problems to authenticate species identifications. Moreover, they suggest that thousands of DNA reads would be needed for proper ancient DNA authentication. This is why we relied on stringent authentication criteria to identify the eukaryotic DNA reads.

In conclusion, we found that the chewed pitch material is an excellent source of ancient DNA, which can be used to understand diet, raw material use, and oral health conditions in prehistoric and early modern populations. We reconstructed several ancient bacterial genomes and found notable amounts of oral pathogens. Furthermore, we compared the microbial profile in chewed pitch mastic particles to modern human salivary microbiomes. Our results show that the bacterial profile is similar to modern periodontitis cases.

The samples also contained eukaryotic species like hazelnut, trout, mallard, red fox, limpet, and red deer. These species provide sources for reconstructing Mesolithic diet and paleoenvironment, while the presence of many of the species identified in this study is supported by archaeological finds. These DNA data may provide clues to specific dietary items but do not represent the whole variety of food sources used during the Scandinavian Mesolithic. They do, however, indicate what members of this particular group had been orally processing before or during their interaction with the pitch.

Our results portray a group of Mesolithic hunter-gatherers in south-western Scandinavia, where individuals likely suffered from dental diseases and were utilising resources from different domains through oral manipulation.

## Materials and methods

### The specific environmental/history/collection context

The Huseby Klev materials were unearthed and collected by archaeologists (including two of the co-authors of this article) during the excavation of this coastal hunter-fisher-gatherer site in the 90s^50^.

The material assemblage was rich and well preserved: human bones, animal bones, plant remains and pieces of masticated pitch were found. The exceptional preservation was presumably caused by a layer of clay which sealed the site into a geological time capsule. Pitch or “tar” pieces are a rather common find at Stone Age sites in Eurasia, and particularly in Scandinavia, where well preserved pieces of masticated pitch are found dating well into the Iron Age^57^. The pitch pieces were produced from birch bark tar, thus making birch a valuable resource during the entire Stone Age. There are about 90 masticated pitch pieces from the site, while tooth imprints in six of them have been cast and osteologically analysed. All the analysed pieces had imprints suggesting individuals younger than 20 years: three aged between 5 and 11 and three teenagers^50^. The pieces we used for the study were directly sampled from the material collection, which is under the guardianship of Bengt Nordqvist.

### Experimental procedure

The DNA extraction and library building was performed at Stockholm University, AFL, in ancient DNA dedicated facilities, all designed to have positive pressure, separate airlock, HEPA filters for the incoming air, UV lights at the ceiling, and constant sodium 3% hypochlorite disinfection (NaOCl). Sampling of the masticated pitch pieces was performed in a “drill laboratory”, a separate room to the “main laboratory” where exclusively sampling of the materials was undertaken to minimise contamination. The material was irradiated in a UV oven at about 6 J/cm2 at 254 nm. When the necessary amount of the samples (between 50 and 100 mg) were collected into 2 mL screw cap tubes, the tubes were taken to the main laboratory for further extraction and library building. A blank was added at this stage. We checked the DNA concentration after extraction using Qubit 3.0 high sensitive double strand DNA fluorometric assay (Thermo Fisher Scientific). We did not observe any trace of contaminating DNA in the blank extractions.

In the main lab extraction was performed using two methods: the Yang extraction method^58^ which is commonly used for extraction of bone tissue (incubation at 55 °C in 1000 μL of buffer per sample containing 0.5 M EDTA pH8, 1 M Urea, and 10 μL of Proteinase K 10 mg/mL, followed by concentration of the supernatant using Amicon membrane filters and purification with MinElute spin columns to obtain 110 μL of final product) and the QIAamp PowerFecal DNA Kit (Qiagen) with which we obtained 100 μL of final product, following the instructions of the kit with minor adjustments.

The libraries were also built in the main laboratory, following a modified protocol by Meyer and Kircher^59^ for double-stranded blunt-end-repair libraries and damage-repaired libraries as in^56^ pre-treating the extracts with USER enzyme. Libraries were amplified with 10 µM index primers, later double-indexed, using AmpliTaq Gold 1000 Units 5 U/μL (Applied Biosystems) for blunt-end libraries, and AccuPrime™ Pfx DNA Polymerase (2.5 U/μL) (Invitrogen) polymerase for damage repair libraries (damage repair applied to only ble004 sample). We determined the number of cycles using quantitative PCR, with reagents from Thermo Scientific and Biomers. Libraries were sequenced on the Illumina Hiseq X platform at the SciLife centre in Stockholm and SciLife centre in Uppsala. The damage repaired libraries of the ble004 sample are shown in Table S12.

### DNA libraries and external datasets

After receiving the sequences, *AdapterRemoval*^60^ was used to trim adapters, nucleotides less than 20 base quality, and filtered shorter than 10 nucleotides and subsequently subjected to human demography pipeline.

In this study we focused on DNA sequences that do not map to the human reference genome. To do this we first filtered human DNA reads from fastq files and we used *cutadapt*^61^ to trim remaining adapters, and nucleotides less than 20 base quality, and 10 nucleotides were also removed. Final sequencing depth of the fastq files is shown in Table S12.

In addition, we used published ancient and modern datasets to understand the microbial composition of ancient mastics. First, we used pre-calculated *MetaPhlAn3* relative abundances of modern human microbiome samples using the *curatedMetagenomicData* package^25–30,62,63^. Next, we downloaded published DNA data from ancient dental calculus and chewed pitch studies^8–11,14–17,19,22–24^. At last, we used published studies that focus on sediments to identify environmental microbiome contribution to pitch samples^28,33,34,64^ (Table S13).

For differential abundance analysis, we used a dataset containing salivary microbiome data of 12 healthy, 12 caries, and 10 periodontitis patients^40,41^.

We downloaded the fastq files from European Nucleotide archive and processed paired-end DNA sequences using *AdapterRemoval* tool^61^ tool by trimming bases that have less than 20 phred quality score and collapsed forward and reverse reads if they had overlapping regions with 11 or more nucleotides.

### Metagenomic profiling of the ancient mastics

We used *MetaPhlAn3* with default options to profile the fastq files of the ancient samples^62^. This tool uses a marker database (that contains approximately 13,500 bacterial and archaeal, 3,500 viral, and 110 eukaryotic marker DNA fragments from ~ 17,000 reference genomes) to profile a DNA library and produces a microbial relative abundance table. This describes the proportion of DNA fragments that belong to a particular taxon, and a sequence alignment map (sam) file that contains the alignment information. After profiling each DNA library, we removed optical PCR duplicates from sam files with *picard-tools*^65^, sam files that correspond to each library were merged for each sample, and relative abundance tables were calculated with *MetaPhlAn3* for each sample.

To further analyse the dataset, we prepared a set of reference and representative genomes of bacterial, archaeal, fungal, and viral species from the NCBI RefSeq database^66^ (N = 6133) and indexed them using the *malt* alignment tool^38^ and we aligned each fastq file to this indexed reference collection with the same tool. We used the *dust-masker*^67^ tool to mask repetitive regions that could create false-positive classifications. Then, we aligned the sequenced libraries to the database using *malt*^38^ with default options, and we obtained binary *rma6* files. These files contain the alignment and taxonomy information for each sequence. Afterwards, we used *MEGAN*^68^ to extract the species absolute abundance values for each fastq file.

### Comparing microbial profiles from ancient samples with modern human microbiome

To understand the metagenomic composition in ancient mastic material, we compared the relative microbial abundances from the ancient mastic samples against a dataset consists of modern human oral cavity (*N*  = 725), modern human nasal cavity (*N*  = 93), modern human skin (*N* = 42), modern human stool (*N* = 500), modern human vagina (*N* = 86), ancient human dental calculus (*N*  = 395), and chewed pitch (*N*  = 1). The site composition of the oral microbiome samples are anterior nares (*N* = 93), buccal mucosa (*N*  = 119), hard palate (*N*  = 1), keratinized gingiva (*N*  = 6), palatine tonsils (*N* = 6), saliva (*N*  = 5), subgingival plaque (*N*  = 72), supragingival plaque (*N*  = 127), and tongue dorsum (*N*  = 198). Some of the oral microbiome samples do not have any site information and therefore were not included in the list.

We calculated pairwise Bray–Curtis distances between each compositional vector using the *vegdist* function in the *vegan* package of the R statistical platform^32,69,70^. The distance matrix was visualised using a non-metric multidimensional scaling (NMDS) implemented in *metaMDS* in the *vegan* library with a K value of 7. The first three axes were selected for visualisation.

At last, we used *SourceTracker*^43^ to quantify the oral microbiome content in chewed pitch materials^30^. To do this, we selected species taxonomic nodes from the *MetaPhlAn3* abundance matrix, and we marked the comparison dataset from modern human oral microbiome samples and ancient dental calculus samples as source materials. Additionally, we included sediment (N = 23), and shallow marine sediment (N = 21) as environmental sources. Then we used the *SourceTracker* tool with the default options, to quantify the source contribution to the ancient samples (Table S14 – S22).

### Generating full genome ancient bacterial sequences

We collected all reads that were assigned to species’ taxonomy nodes and combined them into one fastq file for each species, with all reads that align to at least one reference genome of that species, and nothing else.

Based on the taxonomic assignment results, we selected the corresponding reference genomes (*n* = 281). And we aligned each fastq file to the respective reference genome, separately.

To produce full genome sequences, we extracted aDNA reads for each bacteria using *malt-extract*^38^.

Thereafter, DNA reads for each sample were merged and aligned to their respective bacterial full genome sequences using *bwa*^71^.

Sequence alignment files were filtered using *SAMtools*^72^ with alignment quality (q30) parameter to remove bad alignments, and DNA sequences with length less than 30 nucleotides were discarded from the alignment file. Optical PCR duplicates were removed using *Picard-tools* with the *mark-duplicates* option. Genome coverage was calculated using *SAMtools*^72^. Deamination patterns were obtained by the *mapDamage* tool^73^ with default options. User enzyme treated (damage repaired, *dr*) ble004 libraries were excluded from the analysis to calculate reliable deamination patterns. Results from these libraries are denoted with the suffix *nondr*. Finally, we generated genome authentication plots that contain edit distance distribution, length distribution, deamination patterns, and genome coverage plots. Damage repaired ble004 libraries were added in the analysis after we obtained reliable deamination patterns in non-damage repaired libraries.

Also, we co-assembled DNA reads using the *megahit* tool with the k-list parameter 21,29,39,59,79,99,119^74^. In essence, we de novo assembled all DNA reads that belong to one particular sample and we classified the contigs using *krakenuniq* tool with the full NT database that contains Eukaryotic full length genomes^75,76^. To calculate the authenticity of the contigs, we used *pyDamage* tool^77^. To assess the assembly quality, we used the *Quast* tool^78^ and specified reference genomes of each oral microbe that were used in the reference-based genome assembly step. With this approach we aimed to estimate the assembled proportion of each microbe (Table S2).

### Beta-binomial modelling to find overabundant microbes in ancient pitch materials

A beta-binomial statistical model was used to detect which commensal oral microbes are overabundant in ancient mastics. We first used modern salivary microbiome datasets that contain healthy and dysbiosis cases (periodontitis and dental caries), restricted to the species that are commonly found in human oral cavities, as described in the Human Oral Microbiome Database. Then we used the beta-binomial method implemented in the *corncob*^79,80^ R package to build differential abundance models for each bacterial species. The cases where the differences between healthy and dysbiosis are statistically significant (multiple tests corrected using Benjamini–Hochberg method with a cut-off value of 0.05) suggests that these microbes could be markers of dysbiosis.

After selecting the marker species for dysbiosis in the modern salivary microbiome, we applied the same methodology to ancient mastics. First, the sequencing depth of ancient mastic libraries was scaled so the average depth of each library matched the mean value of the healthy salivary datasets. This way all distributions have the same order of magnitude and are therefore easier to compare.

Then, a beta-binomial test was applied to each sample, and significant taxa and models were extracted. Afterwards, we searched for marker species in the list of significant species, and we restricted the species list to the species in the Human Oral Microbiome Database.

Moreover, the same methodology was used to identify the differences between pitch materials and ancient dental calculus samples that had been identified with periodontitis^17^.

### Machine learning to find predictors of dysbiosis

To estimate the overall health of the people who chewed the pitch material, we used a machine-learning classification method known as random forests^42^. Random forests aggregate predictions from different classification trees to reduce variance and to improve robustness^75^. In essence, the method creates several simple classification trees, each with a randomly chosen subset of features, and combines their predictions through voting.

In our case, the features correspond to the different species considered, and the data is the relative abundance of these species found in each sample. The labels used for training are the dysbiosis conditions: caries, periodontitis, or healthy. In this part, we calculated the relative abundance restricted to oral specific microbial species.

Each random forest is trained using only part of the data (i.e., bagging), and uses the remaining data to evaluate an out-of-bag error rate, which is numerically close to the N-fold classification error rate^76^. In other words, a low out-of-bagging error indicates that the random tree will generalise well for new data.

By evaluating the mean decrease in Gini impurity (MDG), random forests can rank each feature (i.e., species) sorting out which ones are more relevant to characterise the health condition. We used the MDG ranking to iteratively remove the least important feature, until five features remain, following Darst et al.^81^. In each iteration we created ten independent random forests, we averaged the MDG for each feature, and we discarded the feature with the lowest average MDG. This procedure was repeated until five features remained. With this approach, we kept only the features that contribute to the overall accuracy of the model.

This whole feature selection process was repeated three times. Finally, we sorted all the trees built in the feature selection process by their out-of-bagging error rate, and kept the best two models in each major iteration to make a model pool.

To have a simpler interpretation, we handled the periodontitis and caries cases separately, building two independent model pools.

We used the *randomForest* function of the R statistical package with default parameters to build random forests models^70,82^.

Multiple models trained with the same feature set were combined using the *combine* function in the *randomForest* library. This model pool was used to estimate the probability of each health condition for the ancient material, using the *predict* function over the relative abundance tables from the Mesolithic pitch materials. These probability values were then plotted using the *ggplot2* and *ggpubr* libraries on the R statistical package^83,83^.

### Eukaryotic taxonomic classification

To study the presence of eukaryotic species, we first removed the bacterial reads that we identified from the previous step. Then, we used *Kraken2*^84^ to classify these reads using the full NCBI NT database that contains the all available reference sequences at that time.

We also used several confidence thresholds to filter the possible false positive classification (0.1—0.5). We manually restricted the resulting abundance table to keep only eukaryotic taxa with at least 10,000 DNA reads.

To authenticate these eukaryotic DNA reads, we extracted reference sequences for each eukaryote from the Kraken2 database. Then we used *bwa* with custom parameters (L = 16,500, *N* = 0.01, O = 2) to align the reads assigned to each taxa against their corresponding genome^71^.

After alignment, we removed nucleotides shorter than 30 bp, and validated the antiquity of the eukaryotic DNA reads using the same aDNA authentication protocol that we used for bacteria. We plotted the edit distance distribution, length distribution, and deamination plots for each set of DNA reads aligned to a eukaryotic species. Each plot was manually scored between 0 and 1 (1 for ideal and 0 for not ideal) based on the visual confirmation criteria: edit distribution should peak at low values and decline rapidly, there should be deamination patterns close to the reads’ extremes, and the distribution of DNA length should be concentrated in small values. Then, the entries were sorted according to the scores and to the number of DNA reads aligned to the reference genome (Table S7—S11).

## Data and code availability

Next generation sequencing data of ancient mastic materials are available on NCBI Sequence Read Archive via PRJNA994900 Bioproject accession number. The original codes, metagenomic abundance data and final random forests models are published with 10.5281/zenodo.10252965.

### Supplementary Information

Supplementary Information 1.

Supplementary Information 2.
