# Supplementary material for: Metagenomic analysis of Mesolithic chewed pitch reveals poor oral health among stone age individuals
Source: Sci Rep. 2024 Jan 18;14:22125. doi: 10.1038/s41598-023-48762-6 (PMC10796427; doi:10.1038/s41598-023-48762-6)
Supplement: Supplementary file 1 — Supplementary Information 1. [file 41598_2023_48762_MOESM1_ESM.docx]

# **Supplementary Material**

# **Metagenomic analysis of Mesolithic chewed pitch reveals poor oral health among Stone Age individuals**

Emrah Kırdök^1*^, Natalija Kashuba^2^, Hege Damlien^3^, Mikael A. Manninen^4^, Bengt Nordqvist^5^, Anna Kjellström^6^, Mattias Jakobson^7^, A.Michael Lindberg^8^, Jan Stora^6^, Per Persson^3^, Björn Andersson^9^, Andrés Aravena^10^, Anders Götherström^11, 12^

^1^Mersin University, Faculty of Science, Department of Biotechnology Mersin, 33343 Turkey

^2^Uppsala University, Department of Archaeology and Ancient History, Engelska parken, Thunbergsvägen 3H Box 626 751 26 Uppsala, Sweden

^3^Museum of Cultural History, University of Oslo, P.O. Box 6762. St. Olavs Plass NO-0130 Oslo, Norway.

^4^PAES, Ecosystems and Environment Research Programme, Faculty of Biological and Environmental Sciences and Helsinki Institute of Sustainability Science, Viikinkaari 1. P.O. Box 65, University of Helsinki, Finland

^5^Foundation War-Booty Site Finnestorp, Klarinettvägen 75, SE-434 75 Kungsbacka, Sweden

^6^Stockholm University, Faculty of Humanities, Department of Archaeology and Classical Studies, Osteoarchaeological Research Laboratory

^7^Uppsala University, Department of Organismal Biology, Human Evolution, Evolutionsbiologiskt Centrum EBC Norbyvägen 18 A, Uppsala, Sweden

^8^Linnaeus University, Faculty of Health and Life Sciences, Department of Chemistry and Biomedical Sciences, 44018, Hus Vita, Kalmar

^9^Karolinska Insitute, Department of Cell and Molecular Biology (CMB) Karolinska Institutet P.O. Box 285 SE-171 77 Stockholm, Sweden

^10^İstanbul University, Faculty of Science, Department of Molecular Biology and Genetics 34134 Vezneciler, İstanbul, Turkey

^11^Centre for Palaeogenetics, Svante Arrhenius väg 20C, SE-106 91 Stockholm, Sweden

^12^Stockholm University, Faculty of Humanities, Department of Archaeology and Classical Studies, Archaeological Research Laboratory

* Corresponding author: [emrahkirdok@gmail.com](mailto:emrahkirdok@gmail.com)

#

# **Supplementary figures**


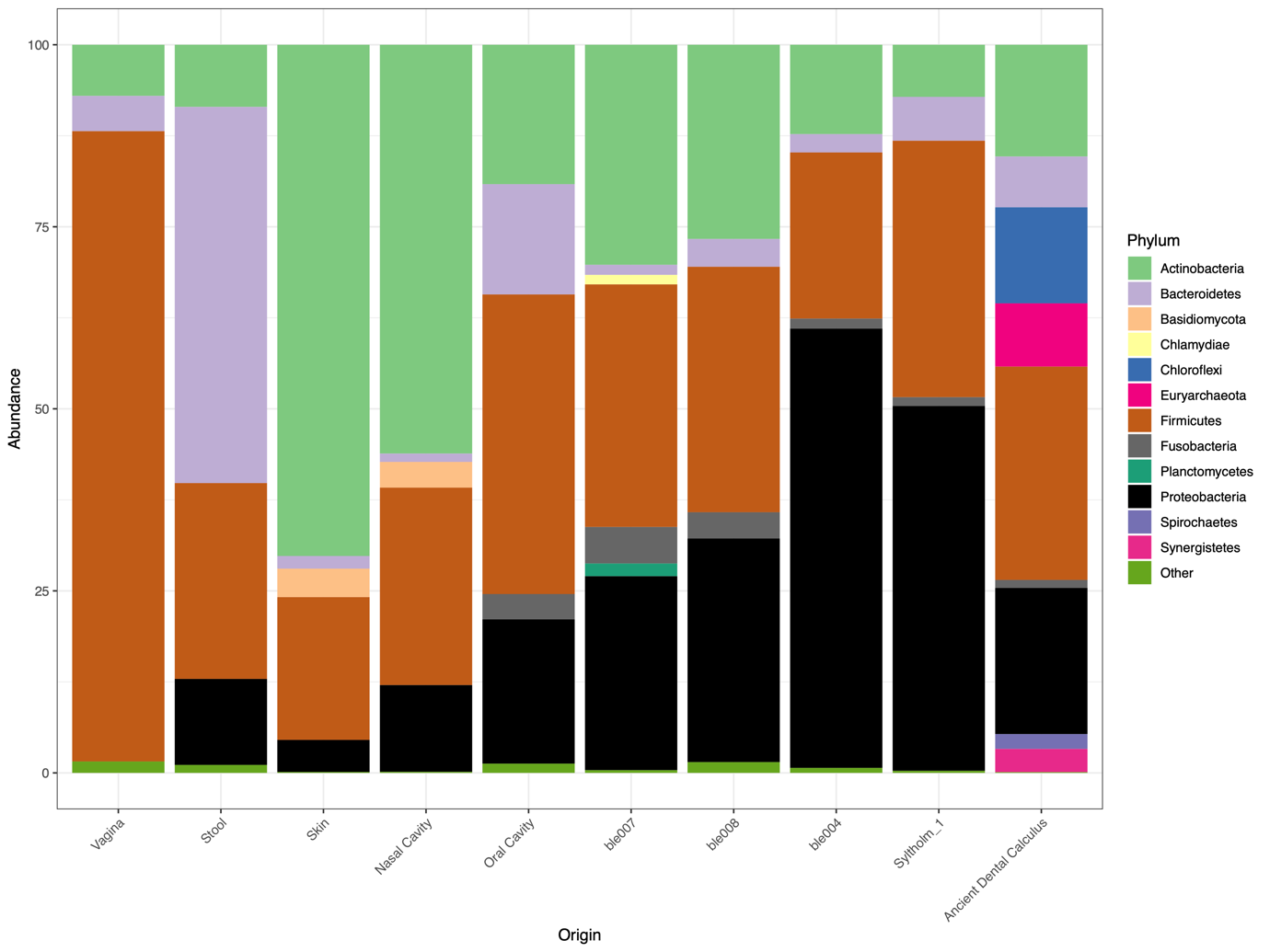


Figure S1: Phylum profiles calculated from the MetaPhlAn3 abundance values show that our ancient pitch pieces and the Syltholm pitch mastic are similar to oral microbial samples Human microbiome project samples show the mean abundance in each group


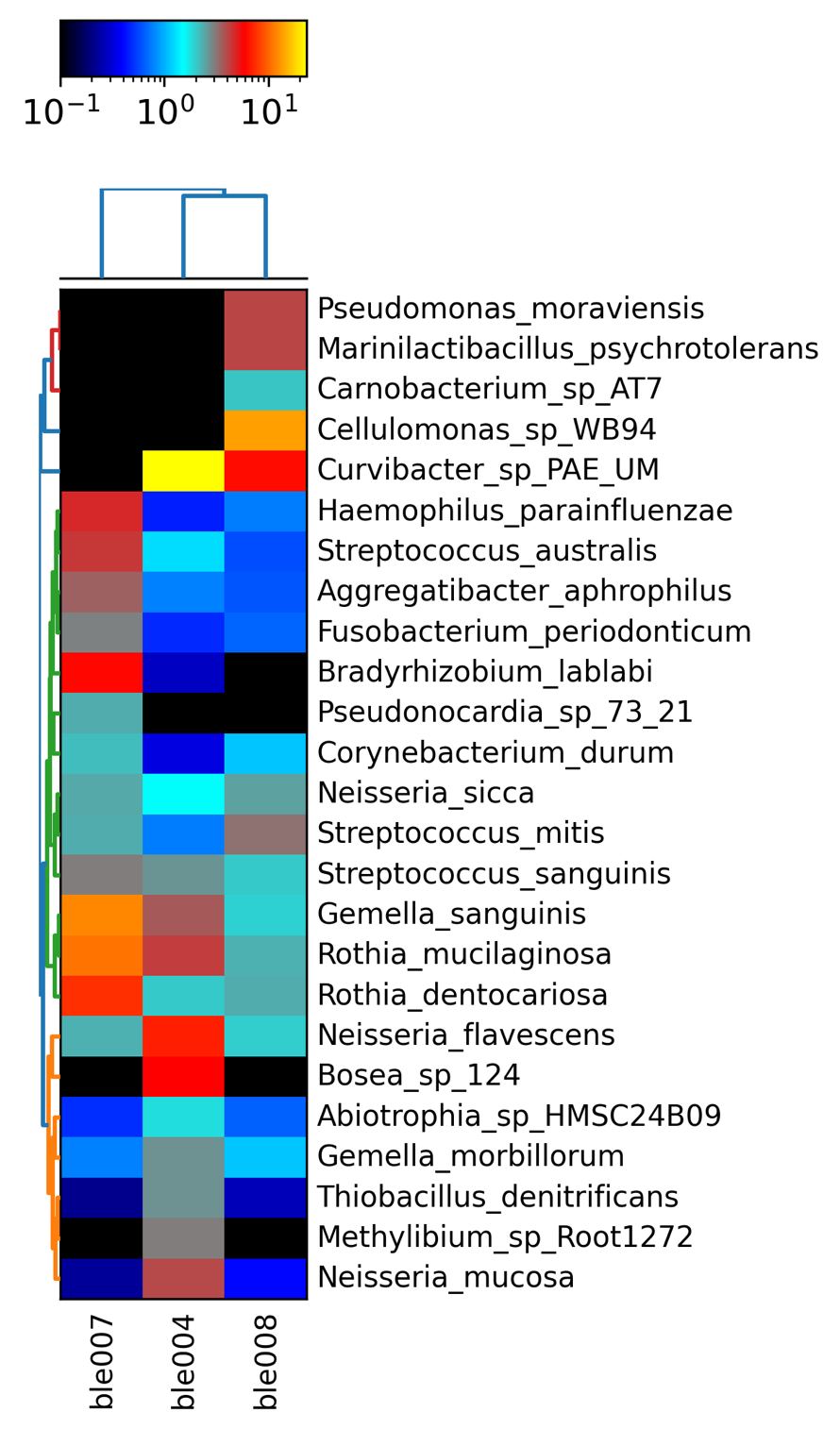


Figure S2: MetaPhlAn3 relative abundance profiles of the first 25 microbes in the ble ancient chewed pitch samples


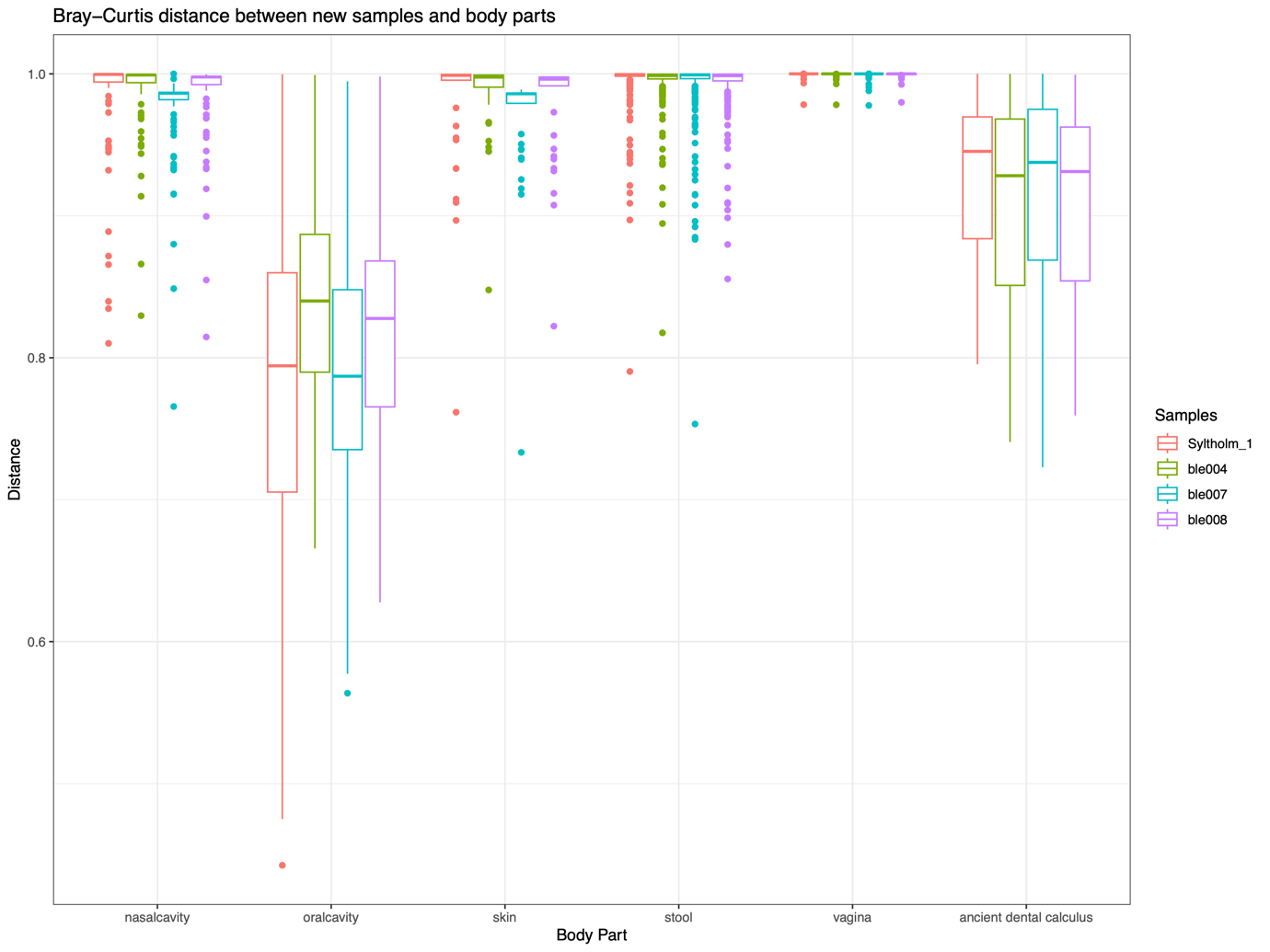


Figure S3: The summary statistics of Bray-Curtis distances of each ancient sample vs body part pair. In overall, chewed pitch pieces have the closest Bray-Curtis values to oral parts of the body.


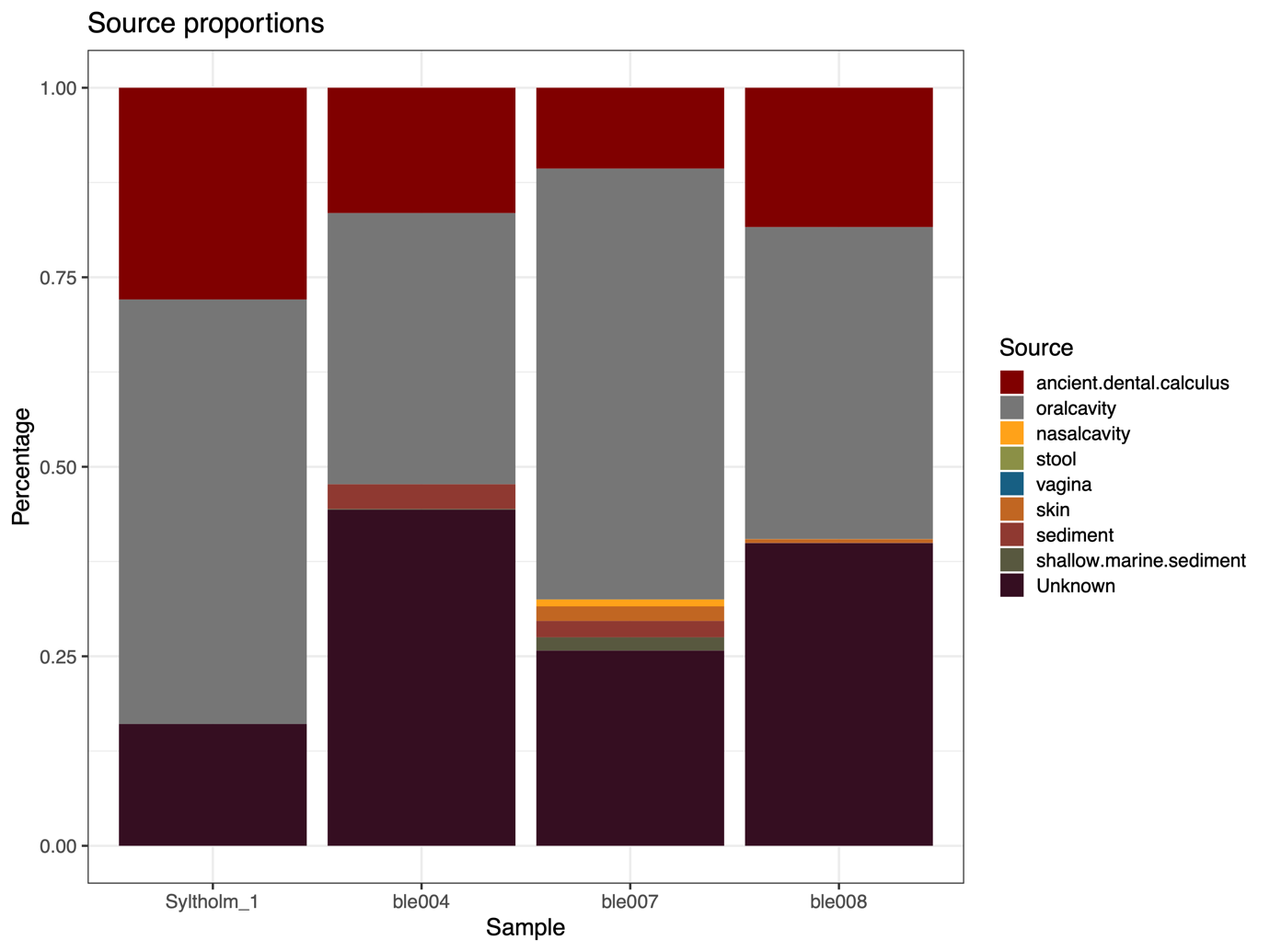


Figure S4: Sourcetracker quantification of Human microbiome and ancient dental calculus contribution to the tested ancient samples.


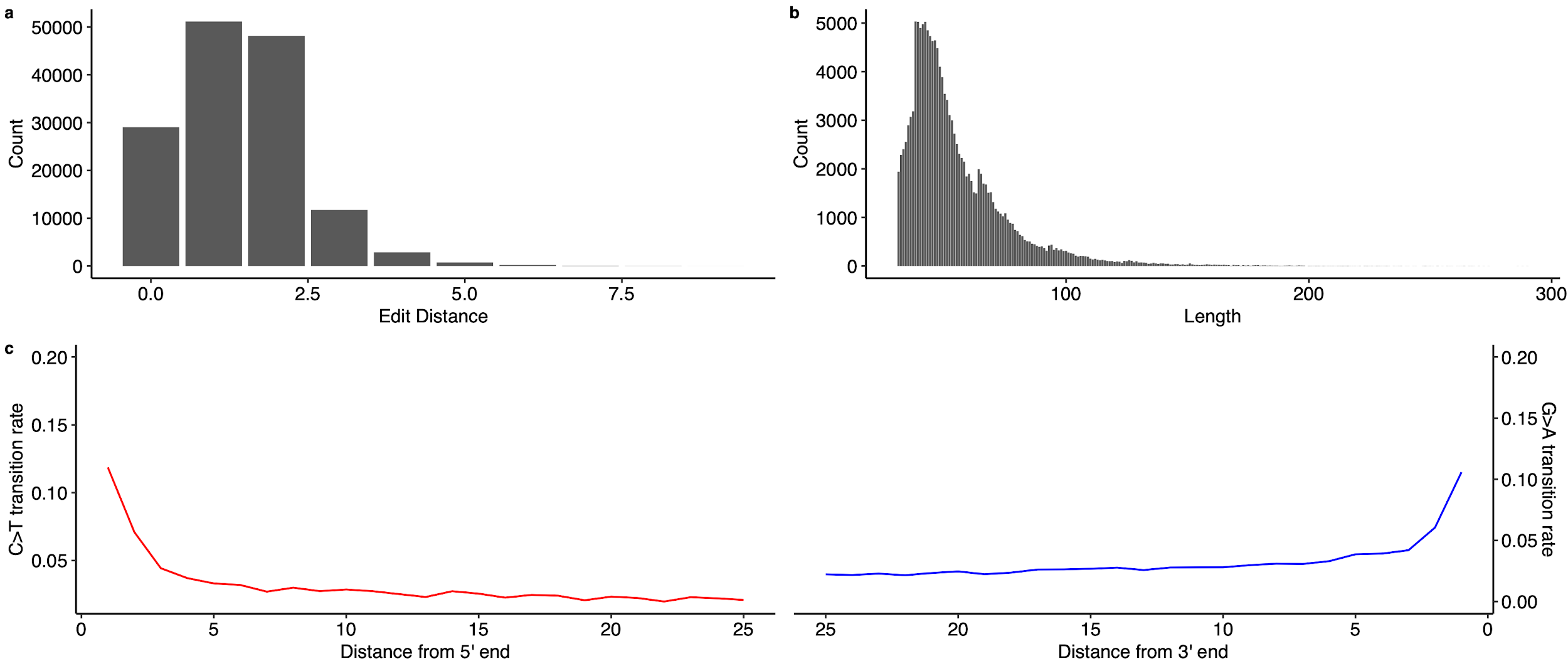


Figure S5: A sample authentication plot for bacterial reads. Three important criteria were sought for authentication: A) Edit distance distribution, B) DNA read length distribution, C) Deamination specific nucleotide substitutions at the 5’ and 3’ ends.

#

# **Supplementary material titles**

Table S1 Reference based alignment analysis of ancient bacteria. First, we aligned DNA reads to their respective reference genomes. Then we applied mapping quality and read length filtering and removed optical PCR duplicates. Finally, we calculated the mean depth of coverage, breadth of coverage and authentication statistics.

Table S2 *De novo* assembly results of the bacterial reads. We used the *megahit* tool to assemble individual ancient bacterial genomes. Genome coverage fraction describes the percent portion of the assembled reference genome.

Table S3 Marker species of Periodontitis and Caries dysbiotic conditions identified by Beta binomial method. In this table, relative abundance fold change (Fold change) values and standard errors (SE) are reported.

Table S4 Beta binomial results of the ancient samples. In this table, relative abundance fold change (Fold change) values and standard errors (SE) are reported.

Table S5 Beta binomial results for ancient chewed pitch materials vs ancient dental calculus samples. In this table, relative abundance fold change (Fold change) values and standard errors (SE) are reported

Table S6 The details of trained Random Forests models. We extracted importance values by using the *importance()* function in the *randomForest* package. In this table, the Mean Decrease Accuracy and Mean Decrease Gini columns describe the feature importances.

Table S7 Authentication of Eukaryotic DNA reads identified by the Kraken2 tool with 0.5 confidence interval threshold. DNA reads identified from each set were aligned to their respective reference genomes and alignment statistics extracted. Alignment statistics were collected before and after the duplication removal step.

Table S8 Authentication of Eukaryotic DNA reads identified by the Kraken2 tool with 0.4 confidence interval threshold. DNA reads identified from each set were aligned to their respective reference genomes and alignment statistics extracted. Alignment statistics were collected before and after the duplication removal step.

Table S9 Authentication of Eukaryotic DNA reads identified by the Kraken2 tool with 0.3 confidence interval threshold. DNA reads identified from each set were aligned to their respective reference genomes and alignment statistics extracted. Alignment statistics were collected before and after the duplication removal step.

Table S10 Authentication of Eukaryotic DNA reads identified by the Kraken2 tool with 0.2 confidence interval threshold. DNA reads identified from each set were aligned to their respective reference genomes and alignments statistics extracted. Alignment statistics were collected before and after the duplication removal step.

Table S11 Authentication of Eukaryotic DNA reads identified by the Kraken2 tool with 0.1 confidence interval threshold. DNA reads identified from each set were aligned to their respective reference genomes and alignment statistics extracted. Alignment statistics were collected before and after the duplication removal step.

Table S12 Total sequencing depth in each fastq file used in this study.

Table S13 Metadata information for published ancient dental calculus, pitch, and sedimentary samples.

Table S14 Prediction of oral cavity contributions to the ancient samples

Table S15 Prediction of ancient dental calculus contributions to the ancient samples

Table S16 Prediction of nasal cavity contributions to the ancient samples

Table S17 Prediction of skin contributions to the ancient samples

Table S18 Prediction of stool contributions to the ancient samples

Table S19 Prediction of vaginal contributions to the ancient samples

Table S20 Prediction of sediment contributions to the ancient samples

Table S21 Prediction of shallow marine sediment contributions to the ancient samples

Table S22 Prediction of unknown contributions to the ancient samples
